# Pathways of antibiotic use in Bangladesh: qualitative protocol for the PAUSE study

**DOI:** 10.1136/bmjopen-2018-028215

**Published:** 2019-01-25

**Authors:** Emily K Rousham, Mohammad Aminul Islam, Papreen Nahar, Patricia Jane Lucas, Nahitun Naher, Syed Masud Ahmed, Fosiul Alam Nizame, Leanne Unicomb

**Affiliations:** 1 Centre for Global Health and Human Development, School of Sport, Exercise and Health Sciences, Loughborough University, Loughborough, UK; 2 Laboratory Sciences and Services Division, International Centre for Diarrhoeal Disease Research, Dhaka, Bangladesh; 3 Paul G Allen School for Global Animal Health, Washington State University, Pullman, Washington, USA; 4 Department of Anthropology, Durham University, Durham, UK; 5 Department of Primary Care and Public Health, Brighton and Sussex Medical School, Sussex University, Brighton, UK; 6 School for Policy Studies, University of Bristol, Bristol, UK; 7 BRAC James P Grant School of Public Health, BRAC University, Dhaka, Bangladesh; 8 Infectious Diseases Division, International Centre for Diarrhoeal Disease Research, Dhaka, Bangladesh

**Keywords:** public health, microbiology, primary care, health policy

## Abstract

**Introduction:**

Global actions to reduce antimicrobial resistance (AMR) include optimising the use of antimicrobial medicines in human and animal health. In countries with weak healthcare regulation, this requires a greater understanding of the drivers of antibiotic use from the perspective of providers and consumers. In Bangladesh, there is limited research on household decision-making and healthcare seeking in relation to antibiotic use and consumption for humans and livestock. Knowledge is similarly lacking on factors influencing the supply and demand for antibiotics among qualified and unqualified healthcare providers.

The aim of this study is to conduct integrated research on household decision-making for healthcare and antibiotic use, as well as the awareness, behaviours and priorities of healthcare providers and sellers of antibiotics to translate into policy development and implementation.

**Methods and analysis:**

In-depth interviews will be conducted with (1) household members responsible for decision-making about illness and antibiotic use for family and livestock; (2) qualified and unqualified private and government healthcare providers in human and animal medicine and (3) stakeholders and policy-makers as key informants on the development and implementation of policy around AMR. Participant observation within retail drug shops will also be carried out. Qualitative methods will include a thematic framework analysis.

A holistic approach to understanding who makes decisions on the sale and use of antibiotics, and what drives healthcare seeking in Bangladesh will enable identification of routes to behavioural change and the development of effective interventions to reduce the health risks of AMR.

**Ethics and dissemination:**

Approval for the study has been obtained from the Institutional Review Board at the International Centre for Diarrhoeal Disease Research, Bangladesh following review by the Research and Ethics Committees (PR-16100) and from Loughborough University (R17-P081). Information about the study will be provided in a participant information letter in Bangla (to be read verbally and given in writing to participants). A written informed consent form in Bangla will be obtained and participants will be informed of their right to withdraw from the study. Dissemination will take place through a 1 day dissemination workshop with key stakeholders in public health and policy, practitioners and scientists in Bangladesh, and through international conference presentations and peer-review publications. Anonymised transcripts of interviews will be made available through open access via institutional data repositories after an embargo period.

**Strengths and limitations of this study**

- The study will provide qualitative insights into antibiotic dispensing and sales practices across a range of qualified and unqualified providers.
- A One Health approach to antimicrobial resistance will be employed to investigate healthcare decision-making for illness in humans and their livestock or animals.
- The study will highlight the healthcare needs of poor and economically disadvantaged groups in Bangladesh.
- As formative research in one rural and one urban site, the sample may not represent all community-level healthcare providers in Bangladesh.

## Introduction

The availability of over-the-counter antibiotics for humans and animals, and a lack of training and compliance with standards among healthcare providers (HCPs) are important contributors to the emergence of antibiotic resistance in Bangladesh and other emerging economies.^1^ Understanding the behaviour and priorities of HCPs, and the needs of consumers is therefore central to developing an effective strategy to tackle antimicrobial resistance (AMR). While there is clear evidence of inappropriate prescribing and use of antibiotics,^1–3^ universal access to healthcare is also an important consideration in countries with inadequate healthcare infrastructure. In this context, retail drug shops and private providers play a critical role in meeting the health needs of the population, particularly among the poorer sectors of society.^4–7^

Bangladesh has an estimated 100 000 licensed retail drug shops and a further 100 000 unlicensed drug shops selling prescription-only drugs including antibiotics.^4^ Unqualified providers are the major providers of healthcare for the poor and disadvantaged.^8^ Within primary healthcare, a high proportion of all consultations result in a prescription for antibiotics: 44% of consultations in Bangladesh^5^ and 70% of consultations in Pakistan^9^ are reported to result in antibiotic prescribing. Within retail drug shops, the majority of antibiotics are purchased without a prescription.^4^ In Tanzania, accredited drug dispensers knew the effects of unnecessary antibiotic use, but their practices were driven by customer demand, habits and profit.^7^ Antibiotics provided to patients without a prescription are likely to be a less appropriate drug, taken for an incorrect duration (course), or of the wrong dose.^10^ Unqualified drug sellers also fall victim to aggressive marketing strategies resulting in overprescribing, multidrug prescribing, using unnecessarily expensive drugs and dispensing drugs without a prescription.^4^

Children and older adults make up a large proportion of people seeking treatment and receiving antibiotics. In Bangladesh, 27% of patients attending rural health centres and 40% at urban centres were under 5 years of age.^5^ Other studies reported that antibiotic prescriptions were most often issued for children aged 0–15 years (35%) followed by adults aged over 60 years (23%).^2^ Polypharmacy, the prescribing of 3 or more drugs, is also common (46% in urban centres, 33% in local health centres).^5^

In response to global actions to reduce AMR, Bangladesh is in the early stages of implementing the National Action Plan on AMR,^11^ has undertaken a situation analysis on AMR containment^12^ and has also developed a Model Pharmacy programme.^13^ These national developments highlight the importance of understanding behaviours that may facilitate or hinder effective implementation and reductions in antibiotic use. Our research can provide valuable insights to support further development of the model pharmacy initiative.

The aim of the study therefore is to take a holistic approach to determine current dispensing, purchase, use and consumption of antibiotics through formal and informal sectors for both humans and animals in Bangladesh.

The specific objectives of the research are:

1. To understand the pathways of antibiotic use within households from the onset of illness to the purchase and consumption of antibiotics.
2. To examine the role of qualified and unqualified private providers and government primary care health service providers in antibiotic prescribing and sales.
3. To inform government policy and identify pathways to behavioural change among HCPs, livestock owners and people or patients who use antibiotics.

## Methods and analysis

### Terminology and definitions of HCPs and sellers of antibiotics

The pluralistic health system in Bangladesh provides multiple avenues of healthcare provision. One system of categorising HCPs is by formal or informal sector provision, whereby informal refers to HCPs who are not registered with a government regulatory authority.^8^ However, this does not make a clear distinction between the different levels of training within the informal sector. For example, family health workers in Bangladesh fall within the informal sector yet are employed by the government/public health system and may undergo significant training. Other providers in the informal sector such as unqualified ‘village’ or ‘quack’ doctors have no formal training. Within retail drug shops, there is also variation in the qualification of providers. In a representative survey in Bangladesh, only 51% of drug shops were staffed by a trained pharmacist with the remaining having no formal training. Of those who were trained, 91% had only the minimum qualification (12-week training certificate).^4^ Based on our pilot work and previous research,^8^ therefore, we identified four main groups of antibiotic providers in human and animal health within the allopathic medical system to reflect the pluralistic health system in Bangladesh.^14^ These are: qualified medical or veterinary graduates; auxiliary medical professionals; semiqualified HCPs and unqualified HCPs (table 1). A range of terms are also used for outlets that sell or dispense antibiotics. For this study, we employ the term ‘drug shop’, operationally defined as ‘a private retail pharmacy or drug store eligible for mandatory approval and registration from the Directorate General of Drug Administration (DGDA)’.^4^ Some literature refers to these outlets in Bangladesh as pharmacies, defined as ‘establishments that sell drugs with or without a doctor’s prescription to the general public*’*.^15^ We use drug shop rather than pharmacy, however, to reflect the individual, privatised and unregulated nature of many retail outlets of antibiotics in Bangladesh.

**Table 1:** Categories of qualified and unqualified healthcare providers involved in prescribing, dispensing or selling antibiotics in Bangladesh

Type of provider	Medical and veterinary	Pharmacy
Qualified graduate practitioner	Bachelor in medicine, bachelor in surgery; doctor of veterinary medicine	Category A pharmacists (MPharm, BPharm)
Auxiliary medical/veterinary professionals	Diploma in medicine or any health-related subject, 1–4 years training	Category B pharmacists (Diploma)
Semiqualified healthcare provider	Less than 1-year training	Category C pharmacists (12-week training certificate)
Unqualified healthcare provider	No health-related training	None

### Study site

The rural sample site for the selection of households and HCPs is in Mirzapur *upazila* (subdistrict) of Tangail district. The urban site is in nearby Tongi *upazila* (subdistrict) of Gazipur district. In both locations, we will select areas that have not been included in any community-based interventions or health surveys. Data collection started in May 2017 and is nearing completion.

### Patient and public involvement

As this is a community-based study, we have not involved patient groups in the study design or research protocol. However, the study team has consulted key personnel in the local government medical and veterinary posts at the rural site (Government Livestock Office and *Upazila* Health Complex). Similarly, in the urban site, we met with the government health department within the Tongi City Corporation to identify appropriate recruitment methods, gain approval to interview government staff and to inform them of the nature of the research. Community representatives were consulted before any recruitment of households to the study.

### Objective 1: pathways of antibiotic use within households

In-depth interviews will collect information on the intrahousehold use of antibiotics including who obtains the antibiotics, on behalf of which family members or livestock; who consumes the antibiotics; how and when antibiotics are obtained and at what cost; for what purpose/illness the antibiotics are used. We will explore beliefs and understandings that drive antibiotic consumption to better understand healthcare seeking decisions; perceptions of susceptibility and health risk, particularly for vulnerable household members; and attitudes towards treatment with antibiotics including beliefs about efficacy and quality. We will establish who makes decisions and what drives healthcare seeking and antibiotic consumption.

### Selection and recruitment of participants for household interviews

We will purposively select households with dependents (children under 5 years and older adults) and livestock (in rural areas) and interview either the household member that makes key decisions around healthcare seeking or the primary caregiver of children and dependents. We will use a combination of random household sampling and discussion with community members to identify households to approach.

We will sample from two socioeconomic strata using a threshold monthly income of less than or greater than Taka 15 000 (approximately GBP150) in rural areas and Taka 20 000 (GBP200) in urban areas, based on the national midrange of household income^16^ to gain insights into socioeconomic influences on decision-making. We will seek maximum variation in the social context of the sample, including purposive sampling of households from the minority ethnic indigenous population in rural areas. Where anyone in the household is unwell with symptoms of diarrhoea, fever or respiratory problems, we will request consent to recontact the household for a follow-up interview to understand how their illness had progressed and what healthcare was sought. We will aim to identify up to 12 households with a currently ill member thereby gaining a longitudinal dimension on the outcomes of health-seeking and treatment. Table 2 provides recruitment targets per strata with a total target sample size of 48 household interviews. This will be reviewed as data collection proceeds in line with qualitative data collection methods and the team’s experience to ensure that data saturation is reached.^17^

**Table 2:** Sampling frame for household interviews about decision-making around healthcare seeking, antibiotic purchasing and consumption

	Rural (total n=24)		Urban (total n=24)	
	<15 000 Taka* (n=12)	>15 000 Taka (n=12)	<20 000 Taka (n=12)	>20 000 Taka (n=12)
Households with child<5 years	9	9	9	9
Households with older family member†	6	6	6	6
Households with a currently ill member	3	3	3	3
Indigenous ethnic households	2	2	0	0

*Monthly household income in Bangladesh Taka.

†Considered as women aged >62 years and men aged >65 years old; eligibility threshold to receive government old age allowance in Bangladesh.

### Objective 2: pathways of antibiotics prescribing and selling by qualified and unqualified HCPs

In-depth interviews with qualified, auxiliary, semiqualified and unqualified HCPs will examine prescribing practices as well as exploring the needs and demands of customers, the influence of medical representatives and drug manufacturers and the need of drug sellers and providers to maintain a livelihood in settings where training may be unavailable or the cost of licensing unaffordable.^4^ We will explore cultural norms and beliefs about the efficacy of antibiotics and knowledge surrounding the development of antibiotic resistance among all groups interviewed.

### Selection and recruitment of HCPs

To maximise diversity, a critical element of qualitative research,^18^ we will sample from four categories of HCPs outlined in table 3. The HCPs will mirror the locations, types of providers (public/government and private) and antibiotic outlets in the study areas. We will recruit community HCPs from central district medical and veterinary facilities, local government facilities, and village-level HCPs in order to capture geographical variation in human and animal medicine providers. The combination of data collection methods will allow triangulation of findings from the different sources of information. A target sample of 46 HCPs will be interviewed to continue until data saturation is reached. This sample strategy is based on a grounded theory approach.^18 19^

**Table 3:** Sampling frame for in-depth interviews with qualified and unqualified healthcare providers

Category	Type of provider	Rural	Urban	Total
Qualified graduate practitioner	Public and private; human and veterinary medicine	5	5	10
Auxiliary medical professionals*	Healthcare workers, paramedics	4	4	8
Semiqualified healthcare provider	Retail drug shop owner or seller	7	7	14
Unqualified healthcare provider	Retail drug shop owner or seller	7	7	14
Total		23	23	46

*Provider in human medicine only, no veterinary equivalent.

### Drug shop observations

Ethnographic observations will be carried out in eight retail drug shops to capture interactions between the antibiotic dispensers or sellers and their clients in a range of settings. Two observation periods, each of 2 hours duration, per outlet will take place in the morning (09:00–11:00) and evening (16:00–18:00) to capture variations in customers at different times of day. The drug shops recruited will include those selling humans medicine, animal medicine and both. As well as observing the seller–customer interactions, we will record the presence of a doctor’s chamber within the premise, whether patients visited/purchased antibiotics themselves, whether customers bought antibiotics with or without prescription and the advice and guidance given to customers.

### Objective 3: key informant interviews and intervention development workshop

#### Policy review and key informant interviews

A desk-based review using systematic search methods will be conducted to identify relevant policy documents and regulations to contain AMR from a One Health perspective. Attention will also be given to regional policy and the situation in Bangladesh in relation to the South and South East Asia regions. Following the review, we will conduct key informant interviews to understand existing incentives to drug shops, the role of the pharmaceutical industry in preventing antibiotic resistance and what information is needed by government to bring about behavioural change. Interviews will take place with Government representatives, pharmaceutical industry and medical representatives, regulatory authority members responsible for licensing and representatives of national or multinational agencies. A total of 35 key informant interviews will be carried out.

#### Informing and developing interventions

Following the formative data collection, a 1 day intervention development workshop will be held to present preliminary research findings, and potential options for intervention or improving current practices around antibiotic sales and use. The workshop will include stakeholders, clinicians (human and animal) and drug shop owners. Based on the analysis of the qualitative assessments, the observations and input from the workshop participants, the study team, in association with a professional communication team, will develop tailored behavioural change messages and materials around the appropriate use of antibiotics. Messages will be produced to incorporate different media including text, audio and video. We will take professional help from a creative agency in the appropriate intervention delivery methods and message development that includes compelling design and depiction.

We will pretest the messages that have been developed for the key target audience(s) to assess their appropriateness and value. Feedback will be collected using an open-ended questionnaire 1 week after delivering the messages in association with the communication professionals. The creative agency, in collaboration with the research team, will revise the pilot communication materials and take this work forward for further research proposals.

### Methodological approaches

Analytical methods will be drawn from behavioural science and social anthropology. The analysis of household decision-making behaviours will highlight the illnesses, symptoms, risk perceptions and household dynamics that lead individuals and carers to seek medical advice and treatment for themselves or others. The views and practices of providers of antibiotics will be explored by applying the ‘social lives of medicines’ theory as an anthropological concept. Whyte *et al* 20 propose that medicines also have social lives that incorporate their production and marketing, their prescription, their distribution through intertwined formal and informal channels, their deaths through one or another form of consumptions and finally their after death in the form of efficacy in modifying bodies. The framework will enable conceptualisation of antibiotic prescribing behaviour and use, and knowledge of antibiotic resistance from the perspective of both providers and household members.

Interviews will be conducted in Bangla, audio recorded and transcribed in full in Bangla. Any identifying characteristics of participants, individuals or private practices will be anonymised.

### Data analysis

#### Translation

The challenges of translation of qualitative research are summed up as: ‘translating the participant’s narrative data, which could yield colloquial source language and nuances, to a professional style in a different language; this may impose difficulties when trying to reduce the gap between different languages.’^21^ On this topic, Twinn^22^ establishes empirically (using a small number of interviews in Chinese or translated into English) that although little difference to the main themes emerges, important differences in the detailed material are apparent, particularly where differences in language structure made translation more challenging. Generally, later translation to English is preferred where most of the researchers are non-English speaking.^23^

In this study, the team comprises English, non-English speaking and some bilingual researchers. Our decision about how much to translate and at what point also reflects the pragmatic constraints of cost and time resources.^24^ For quality assurance reasons, we aim to keep transcription and translation within the core study team as much as possible, ensuring a shared understanding of study aims and approach.^25 26^

Our chosen solution is to translate one-third of transcripts in full into English, by native Bangla researchers. The translated transcripts will reflect the variety of our samples and will be used as the basis for the framework for analysis. The remaining Bangla transcripts will be coded in Bangla, and then the text in the framework will be translated into English. This method will allow the whole team (including non-Bangla speakers) to contribute to the development of the analysis structure and first draft analysis, while allowing the early analyses to be conducted in Bangla.^23 27 28^

#### Analysis

A framework analysis will be applied to the qualitative interview data.^29^ A framework analysis involves case- and theme-based approaches, with matrix display and data reduction through summarisation and synthesis. This method retains links to the original data and the output allows comprehensive and transparent data analysis. The framework analysis will be based on the principle of thematic analysis. This method enables teams to work together to produce a shared (and highly structured) analysis drawing across deductive and inductive elements, lending itself to multidisciplinary health research.^30^ Prior to data collection, we will ensure a good fit between topic guides and the analytic frameworks developed. The framework for analysis will incorporate the key theoretical constructs and respond to the context of policy and practice to include a range of deductive themes. Further themes will be induced from the interview data.

For household interviews, the first draft framework will be drawn up using translated interviews in English then shared with the team for comment and amendment. The researchers will work together charting interviews, comparing their approaches and agreeing the final framework. Following this, all remaining interviews in Bangla will be charted and translated into the frameworks in English. Data familiarisation will be carried out using the data matrices produced by this process.

The interviews with HCPs and key informants will be analysed using the same principles as household interviews and by the same team to identify emerging patterns, higher order themes, examining commonalities and contradictions in the data. The framing components will include a selection of inductive and deductive themes. An appropriate balance of integration between empirical data and interpretation will be maintained. The investigators will extract the meaning of the empirical data and interpret them while acknowledging the complexity of the phenomena of antibiotic use in the context.^19^

## Conclusion

This interdisciplinary study includes public health, policy and social and behavioural science approaches to the challenges of tackling AMR; supported by the AMR Cross-Council Initiative theme ‘AMR Within and Beyond the Healthcare Setting’. An integrated understanding of the behaviours of households, drug sellers and HCPs around the sales, purchase and consumption of antibiotics in rural and urban Bangladesh is needed to inform stakeholders responsible for implementing national actions on antibiotic use and consumption. The expected outputs of the study include formative data on the relationships between households and their local HCPs, motivations for seeking or selling antibiotics for people and animals, and knowledge and awareness of how antibiotics work and what factors lead to antibiotic resistance. The research will give a more comprehensive assessment of the way in which antibiotics are used in human and animal domains. The research will lead towards the development of potential messages and materials for behavioural change communication to complement future interventions on AMR.

## Supplementary Material

Reviewer comments

## References

[1] WHO. Worldwide country situation analysis: response to antimicrobial resistance. 2015 http://www.who.int/drugresistance/documents/situationanalysis/en/ (Accessed 25 Nov 2018).

[2] Biswas M, Roy MN, Manik MI, et al Self medicated antibiotics in Bangladesh: a cross-sectional health survey conducted in the Rajshahi City. BMC Public Health 2014;14:847 10.1186/1471-2458-14-847 25124712 PMC4150958

[3] Mohiuddin M, Rashid SF, Shuvro MI, et al Qualitative insights into promotion of pharmaceutical products in Bangladesh: how ethical are the practices? BMC Med Ethics 2015;16:80 10.1186/s12910-015-0075-z 26625723 PMC4666091

[4] SIAPS. Baseline study of private drug shops in Bangladesh: findings and recommendations. 2015 http://siapsprogram.org/publication/altview/baseline-study-of-private-drug-shops-in-bangladesh-findings-and-recommendations/english/ (Accessed 25 Nov 2018).

[5] Ahmed SM, Islam QS Availability and rational use of drugs in primary healthcare facilities following the national drug policy of 1982: is Bangladesh on right track? J Health Popul Nutr 2012;30:99–108. 10.3329/jhpn.v30i1.11289 22524126 PMC3312366

[6] Bloom G, Wilkinson A, Tomson G, et al Addressing Resistance To Antibiotics In Pluralistic Health Systems. Brighton: STEPS Centre, 2015.

[7] Dillip A, Embrey M, Shekalaghe E, et al What motivates antibiotic dispensing in accredited drug dispensing outlets in Tanzania? A qualitative study. Antimicrob Resist Infect Control 2015;4:30 10.1186/s13756-015-0073-4 26199723 PMC4509560

[8] Ahmed SM, Hossain MA, Chowdhury MR Informal sector providers in Bangladesh: how equipped are they to provide rational health care? Health Policy Plan 2009;24:467–78. 10.1093/heapol/czp037 19720721

[9] Joseph HA, Agboatwalla M, Hurd J, et al What happens when “germs don’t get killed and they attack again and again”: perceptions of antimicrobial resistance in the context of diarrheal disease treatment among laypersons and health-care providers in Karachi, Pakistan. Am J Trop Med Hyg 2016;95:221–8. 10.4269/ajtmh.15-0661 27139438 PMC4944694

[10] Morgan DJ, Okeke IN, Laxminarayan R, et al Non-prescription antimicrobial use worldwide: a systematic review. Lancet Infect Dis 2011;11:692–701. 10.1016/S1473-3099(11)70054-8 21659004 PMC3543997

[11] Ministry of Health & Family Welfare: Disease Control Unit. Antimicrobial resistance containment in Bangladesh 2017-2022. 2017 http://www.who.int/antimicrobial-resistance/national-action-plans/library/en (Accessed 25 Nov 2018).

[12] The GARP-Bangladesh National Working Group. Antibiotic use and resistance in Bangladesh: situation analysis and recommendations on antibiotic resistance: Directorate General of Drug Administration, 2018 https://cddep.org/publications/bangladesh-situation-analysis-amr/ (Accessed 25 Nov 2018).

[13] Government of Bangladesh Ministry of Health & Family Welfare. Standards for the establishment and operations of model pharmacies and model medicine shops. 2016 http://www.dgda.gov.bd/index.php/2013-03-31-05-16-29/guidance-documents/175-guideline-for-model-pharmacy (Accessed 25 Nov 2018).

[14] Nahar P Health seeking behaviour of childless women in Bangladesh: an ethnographic exploration for the special issue on: loss in child bearing. Soc Sci Med 2010;71:1780–7. 10.1016/j.socscimed.2010.07.026 20728974

[15] Adams AM, Ahmed T, El Arifeen S, et al Innovation for universal health coverage in Bangladesh: a call to action. The Lancet 2013;382:2104–11. 10.1016/S0140-6736(13)62150-9 24268605

[16] Bangladesh Bureau of Statistics, Ministry of Planning. Report on the Household Income and Expenditure Survey 2010. 2011 http://catalog.ihsn.org/index.php/catalog/2257 (Accessed 25 Nov 2018).

[17] Guest G, Bunce A, Johnson L How many interviews are enough?: an experiment with data saturation and variability. Field Methods 2006;18:59–82. 10.1177/1525822X05279903

[18] Patton MQ Qualitative research & evaluation methods: integrating theory and practice. 4 edn: Sage Publications Inc, 2015:832.

[19] Green J, Thorogood N Qualitative methods for health research. 4th edn London: Sage Publications Ltd, 2005.

[20] Whyte SR, van der Geest S, Hardon A Social lives of medicine. Cambridge: Cambridge University Press, 2002:3–19.

[21] Al-Amer R, Ramjan L, Glew P, et al Translation of interviews from a source language to a target language: examining issues in cross-cultural health care research. J Clin Nurs 2015;24:1151–62. 10.1111/jocn.12681 25181257

[22] Twinn S An exploratory study examining the influence of translation on the validity and reliability of qualitative data in nursing research. J Adv Nurs 1997;26:418–23. 10.1046/j.1365-2648.1997.1997026418.x 9292378

[23] Santos HP, Black AM, Sandelowski M Timing of translation in cross-language qualitative research. Qual Health Res 2015;25:134–44. 10.1177/1049732314549603 25189538

[24] Hendrickson SG, Harrison TC, Lopez NA, et al Translation cost, quality, and adequacy. J Nurs Scholarsh 2013;45:185–91. 10.1111/jnu.12021 23676081 PMC3674223

[25] Easton KL, McComish JF, Greenberg R Avoiding common pitfalls in qualitative data collection and transcription. Qual Health Res 2000;10:703–7. 10.1177/104973200129118651 11066874

[26] Lopez GI, Figueroa M, Connor SE, et al Translation barriers in conducting qualitative research with Spanish speakers. Qual Health Res 2008;18:1729–37. 10.1177/1049732308325857 19008363

[27] Shimpuku Y, Norr KF Working with interpreters in cross-cultural qualitative research in the context of a developing country: systematic literature review. J Adv Nurs 2012;68:1692–706. 10.1111/j.1365-2648.2012.05951.x 22420685

[28] Helmich E, Cristancho S, Diachun L, et al ‘How would you call this in English?’: Being reflective about translations in international, cross-cultural qualitative research. Perspect Med Educ 2017;6:127–32. 10.1007/s40037-017-0329-1 28220459 PMC5383565

[29] Spencer L, Ritchie J, Ormston R, Analysis principles and processes : Ritchie J, Lewis J, McNaughton Nicholls C, Barnard M, Ormston R, *et al* Qualitative research in practice: a guide for social science students and researchers. London: Sage Publications Ltd, 2013:269–94.

[30] Gale NK, Heath G, Cameron E, et al Using the framework method for the analysis of qualitative data in multi-disciplinary health research. BMC Med Res Methodol 2013;13:117 10.1186/1471-2288-13-117 24047204 PMC3848812

