# Supplementary material for: Pathways of antibiotic use in Bangladesh: qualitative protocol for the PAUSE study
Source: BMJ Open. 2019 Jan 25;9(1):e028215. doi: 10.1136/bmjopen-2018-028215 (PMC6352800; doi:10.1136/bmjopen-2018-028215)
Supplement: Reviewer comments [file bmjopen-2018-028215.reviewer_comments.pdf]

PEER REVIEW HISTORY

BMJ Open publishes all reviews undertaken for accepted manuscripts. Reviewers are asked to complete a checklist review form (**http://bmjopen.bmj.com/site/about/resources/checklist.pdf**) and are provided with free text boxes to elaborate on their assessment. These free text comments are reproduced below.

ARTICLE DETAILS

**TITLE (PROVISIONAL)**	Pathways of antibiotic use in Bangladesh: a qualitative protocol for the PAUSE study
**AUTHORS**	Rousham, Emily; Islam, Mohammad; Nahar, Papreen; Lucas, Patricia; Nahar, Nahitun; Ahmed, Syed Masud; Nizame, Fosiul; Unicomb, Leanne

This article was not externally reviewed at BMJ Open. Protocols that have been independently assessed prior to submission to BMJ Open are usually fast-tracked to publication on the grounds that further substantial changes will not be possible. This independent assessment will usually be external, independent review for both a substantial grant award from a non-commercial or government funder and ethics approval.
